# Her-2 Expression in Gastroesophageal Intestinal Metaplasia, Dysplasia, and Adenocarcinoma

**DOI:** 10.1097/PAI.0000000000000243

**Published:** 2016-10

**Authors:** Khaldoun Almhanna, Marilin Rosa, Evita Henderson-Jackson, Kun Jiang, Rania Shamekh, Zena Sayegh, Mokenge P. Malafa, Domenico Coppola

**Affiliations:** * Department of Gastrointestinal Oncology, H. Lee Moffitt Cancer Center, Research Institute; † Department of Anatomic Pathology, H. Lee Moffitt Cancer Center, Research Institute; ∥ Department of Chemical Biology and Molecular Medicine, H. Lee Moffitt Cancer Center, Research Institute; ¶ Department of Tumor Biology, H. Lee Moffitt Cancer Center, Research Institute; § Department of Pathology and Cell Biology, University of South Florida, Tampa, FL; ‡ Department of Oncological Sciences, University of South Florida, Tampa, FL

**Keywords:** advanced esophageal cancer, Her-2, ToGA

## Abstract

Overexpression of human epidermal growth factor receptor 2 protein (Her-2) in Barrett neoplasia is significant for targeted therapy with trastuzumab. Here, we studied the frequency of Her-2 overexpression in Barrett adenocarcinoma and precursor lesions. Retrospective formalin-fixed paraffin-embedded tissue samples of 25 normal (NM) esophageal mucosa, 50 Barrett esophagus (BE) without dysplasia, 49 BE with low-grade dysplasia (LGD), 50 BE with high-grade dysplasia (HGD), and 50 invasive adenocarcinoma (ICA) were used. A BE tissue microarray was built and analyzed by Her-2 immunohistochemistry (IHC) and Her-2 dual in situ hybridization (DISH). Her-2 IHC expression was negative in NM and low in 26% of BE (IHC score: 1+) and in 24.5% of LGD (IHC score: 1 to 2+). Her-2 overexpression was seen in 28% of HGD and in 24% of ICA (IHC score: 2 to 3+). Her-2 DISH was negative in NM and BE but positive in 6% of LGD, 20% of HGD, and 18% of ICA. Differences in Her-2 DISH positivity between NM and HGD or ICA were statistically significant (*P* = 0.02), but those between NM and LGD or HGD and ICA were not (*P* = 0.2). Although Her-2 overexpression results in ICA were similar to previous reports, the finding of 28% in HGD was unexpected and may have clinical implications. Positive Her-2 DISH in 6% of LGD is novel, suggesting a role of Her-2 during BE progression.

The incidence of esophageal squamous cell carcinoma is decreasing in the United States; however, the incidence of adenocarcinoma arising from Barrett esophagus (BE) is increasing dramatically.^1^ In 2014, there were 18,170 cases of esophageal cancer in the United States, with 15,450 patients expected to die from the disease.^1^

BE is believed to be a consequence of acid reflux. It has been suggested that acid reflux may introduce mutations in esophageal cells due to acid pH-induced DNA damage.^2^ However, acid reflux can also cause inflammatory changes known to contribute to carcinogenesis.^3^ Despite significant improvements in understanding its disease biology, the 5-year survival rate for esophageal cancer remains low. Targeted agents have failed to add any meaningful survival benefit in this patient population despite promising preclinical data. Recently, however, the Trastuzumab for Gastric Cancer (ToGA) trial showed improvement in patients who received trastuzumab plus chemotherapy versus chemotherapy alone (median overall survival of 13.8 mo compared with 11.1 mo, with hazard ratio 0.74; 95% confidence interval, 0.60–0.91; *P* = 0.0046).^4^ Response rate, time to progression, and duration of response were also significantly higher in the trastuzumab plus chemotherapy group.^4^

Human epidermal growth factor receptor 2 protein (Her-2) is a member of the *EGFR* family. The *Her-2* oncogene encodes for a 185-kDa transmembrane glycoprotein receptor with intracellular tyrosine kinase activity.^2^ Her-2 is a cell membrane surface-bound receptor tyrosine kinase and is involved in signal transduction leading to cell growth and differentiation. The *Her-2* gene is a proto-oncogene, and it is located on the long arm of human chromosome 17.^5,6^

Amplification of *Her-2* has been described in tissue samples from different malignancies such as breast cancer, gastric cancer, and ovarian cancer.^3,7^ Investigations have also correlated Her-2 overexpression with poor prognosis in patients with ovarian and breast cancer.^8^

The guidelines for reporting and interpreting Her-2 overexpression/amplification in breast cancer have been well established for more than a decade, and trastuzumab and several other anti-Her-2–targeted treatments are routinely used in this patient population. Guidelines for standardized and validated Her-2 immunohistochemical testing in gastric and esophageal cancer have recently been proposed.^9^ Hofmann et al^10^ proposed a modified Her-2 scoring system in gastric cancer. Their study showed a moderate to strong membranous Her-2 staining pattern and higher rate of Her-2 tumor heterogeneity in gastric adenocarcinoma as compared with breast cancer.^10^ In esophageal cancer and its precursor lesions including BE, data on Her-2 amplification and overexpression frequency are limited and correlation investigations between Her-2 amplification and prognosis have shown conflicting results.^11–13^

In this study, our aim was to investigate the frequency of Her-2 overexpression and amplification in esophageal adenocarcinoma and in its precursor lesions using immunohistochemistry (IHC) and dual in situ hybridization (DISH).

## MATERIALS AND METHODS

### Patients

Upon Institutional Review Board approval, the clinical records and histologic specimens from 425 patients were reviewed to select 50 cases of BE without dysplasia, 49 cases of BE with low-grade dysplasia (LGD), 50 cases of BE with high-grade dysplasia (HGD), and 50 cases of invasive adenocarcinoma (ICA). The tissues were selected from endoscopic mucosal resection or esophagogastrectomy resection specimens. The cases were not paired dysplasia and adenocarcinoma. Samples of NM gastroesophageal mucosa were selected from an area near the resection margins and at least 10 cm away from the tumor. This could be obtained from only 25 of the selected specimens. The clinical pathologic features of the tumors included in the study are listed in Table 1.

### Pathologic Evaluation

The hematoxylin and eosin (H&E) slides were evaluated by 2 pathologists with expertise in gastrointestinal pathology (K.J. and D.C.) to confirm the pathologic diagnosis. The pathologists selected slides with obvious areas showing BE, LGD, HGD, and ICA. The formalin-fixed paraffin-embedded tissue blocks corresponding to the H&E slides selected were pulled from the pathology files and were used to produce a Barrett esophagus tissue microarray (BE-TMA). Serial 3-μm sections were cut from the BE-TMA to perform Her-2 IHC and DISH studies to assess Her-2 and chromosome 17 numerical alterations. The first and last sections of each series were stained with H&E. The IHC Her-2 expression was scored following the guidelines used for the ToGA study^4^ as follows: (1) no staining or no membranous staining of tumor cells was scored as “0”; (2) tumor cells with faint membrane staining irrespective of percentage of tumor cells were scored as “1+”; (3) tumor cells with weak to moderate membrane staining irrespective of percentage of tumor cells were scored as “2+”; (4) tumor cells with strong complete, basolateral, or lateral membrane reactivity irrespective of percentage of tumor cells were scored as “3+.”^8^

### TMAs

For each of the selected slides, the corresponding formalin-fixed paraffin-embedded tumor block was pulled from the H. Lee Moffitt Cancer Center pathology files and used to construct a BE-TMA. The BE-TMA was composed of 235 (1 mm) cores of tissue representing 25 NM, 50 BE, 49 LGD, 50 HGD, and 50 ICA and controls.

### IHC

The use of BE-TMA in our study allowed the entire cohort (NM, BE, LGD, HGD, and ICA) to be analyzed in 1 batch on only 1 slide. Appropriate positive and negative controls were used. The BE-TMA was stained with rabbit monoclonal antibody (4B5) (Ventana Medical Systems, using the Benchmark XT). Her-2 protein expression by IHC evaluation was based on the modified scoring criteria established for gastric/gastroesophageal junction cancer and the ToGA trial.^4,9,10^ For the purposes of our study, which used 1-mm cores of tissue representing NM, BE, LGD, HGD, and ICA, with scoring as described above. The Her-2-stained TMA was examined by 2 independent observers (E.H.-J., D.C.), and a consensus score was reached for each specimen.

### DISH

*Her-2* gene amplification by in situ hybridization was performed using the Ventana INFORM Her-2 Dual ISH DNA Probe Cocktail assay (Ventana Benchmark Ultra Platform). This assay detects the *Her-2* gene using a dinitrophenyl-labeled probe and the chromosome 17 centromere using a digoxigenin-labeled probe. The *Her-2* gene was visualized as a discrete black signal, using the Ventana ultraview silver ISH dinitrophenyl (SISH). The chromosome 17 centromere was visualized as a red signal using the Ventana ultraview Red ISH digoxigenin detection (Red ISH). Using a ×40 or ×60 objective, analysis of ISH was performed on areas of NM squamous mucosa, BE, LGD, HGD, and ICA. For each nucleus, we conducted a manual count of the number of Her-2 signals and the number of centromere 17 (CEP17) signals using bright-field microscopy. The ratio of the average number of *Her-2* gene copies to the average number of Chr17 copies was calculated. Indeterminate results were either due to absence of target cells, no tissue core present, unacceptable nuclear morphology (unable to distinguish NM cells from target cells), unacceptable background (SISH “dust”), or weak/absent ISH staining in target cells. For our analysis, a Her-2-to-Chr17 ratio of <2.0 was considered DISH-negative, whereas a Her-2-to-Chr17 ratio ≥2.0 was considered DISH-positive.

### Statistical Analysis

The associations between *Her-2* gene amplification/Her-2 protein overexpression, chromosome 17 aneusomy, and the presence of BE, BE dysplasia (LGD or HGD), and ICA were evaluated by Pearson χ^2^ tests, with *P* < 0.05 considered significant. Histology was categorized at 3 levels: BE with LGD, BE with HGD, and ICA. IHC for Her-2 protein was categorized at 4 levels based on the Food and Drug Administration’s (FDA’s) approved scoring system (0, 1, 2, 3); DISH for *Her-2* gene was considered positive when amplified and negative when not amplified.

## RESULTS

### Clinicopathologic Characteristics

Patients characteristics are shown in Table 1. The cases of ICA included 47 male and 3 female. The tumor size ranged between 0.2 and 7.3 cm (median, 2.1 cm). Five tumors were well differentiated, 23 were moderately differentiated, and 22 were poorly differentiated. Twenty-six cases were stage I, 14 cases were stage II, 9 cases were stage III, and 1 case was stage IV. Only 10 cases had received preoperative adjuvant radiochemotherapy.

### Immunohistochemical Results

**FIGURE 1.**
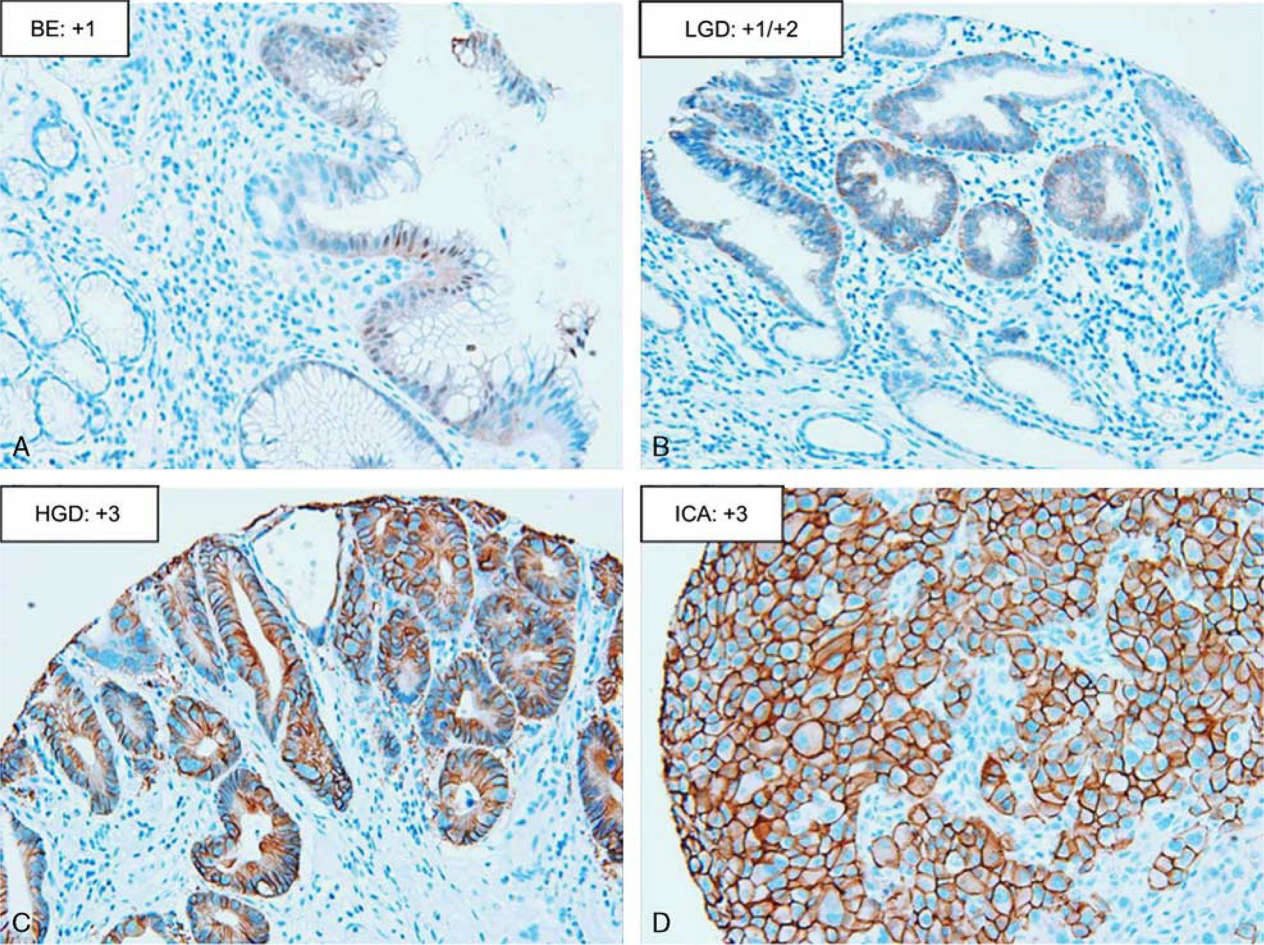
Immunohistochemistry of Her-2 in (A) Barrett esophagus (BE), (B) low-grade dysplasia (LGD), (C) high-grade dysplasia (HGD), and (D) invasive adenocarcinoma (ICA). The stain was evaluated using the modified scoring criteria for gastric/gastroesophageal junction cancer, based on Trastuzumab for Gastric Cancer trial.

All NM were negative for Her-2 (IHC score: 0). BE showed low expression of Her-2 (IHC score: 1+) in 13/50 (26%) of cases. In LGD, Her-2 overexpression was found in 12/49 (24.5%) of cases (IHC score: 1+). In HGD and in ICA, we found overexpression of Her-2 (IHC score: 2–3+) in 14/50 (28%) and 12/50 (24%) of cases, respectively (Figs. 1A–D). Differences in IHC scores between NM or BE and HGD and ICA were statistically significant (*P* = 0.02).

### DISH Results

The DISH results demonstrated a similar pattern of *Her-2* overexpression (ratio >2). NM and BE showed no *Her-2* amplification (0/25 and 0/50 cases, both 0%, respectively). In LGD, *Her-2* was amplified in 3/50 (6%) of cases. In HGD and ICA, *Her-2* amplification was seen in 10/50 (20%) and 9/50 (18%) of cases.

These results show statistically significant differences in IHC expression between NM or BE and HGD or ICA (*P* < 0.05). In contrast, the IHC expression between NM and BE or HGD and ICA was not statistically significant (*P* > 0.05). The differences in *Her-2* DISH amplification between NM or BE and HGD or ICA were also statistically significant (*P* = 0.02), whereas those between NM and LGD or HGD and ICA were not (*P* = 0.2).

**FIGURE 2.**
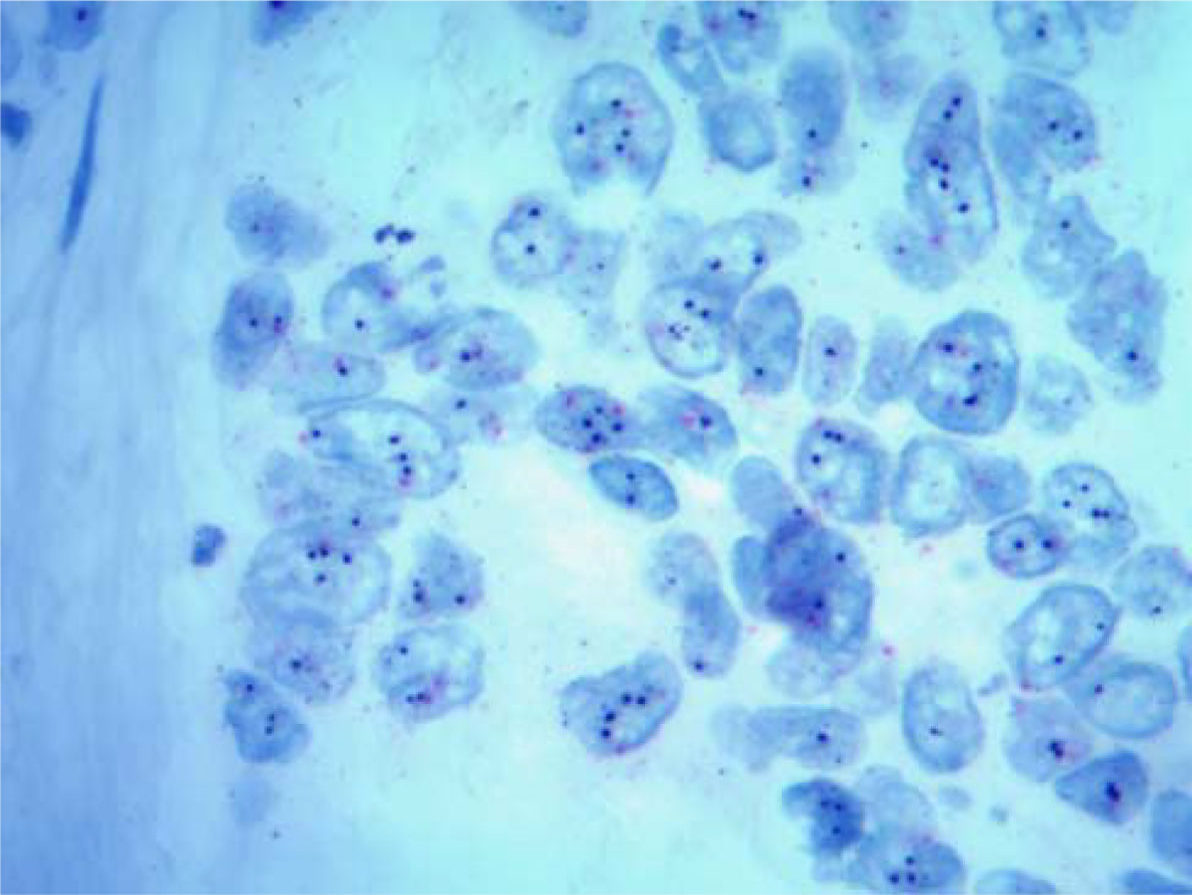
Dual in situ hybridization analysis of a case of invasive adenocarcinoma showing amplification.

All amplified cases were due to increased copy number of Her-2 signals per cell. No cases of monosomy of chromosome 17 or coamplification were observed (Fig. 2). The DISH results are shown in Table 2.

### Subset of Cases With IHC and DISH Performed on Whole-Tissue Sections

Eleven representative cases were selected to evaluate Her-2 results using IHC and DISH in whole-tissue section. With this approach, we aimed to evaluate the presence of tumor heterogeneity as the use of only TMA does not allow adequate assessment of tumor heterogeneity.

**FIGURE 3.**
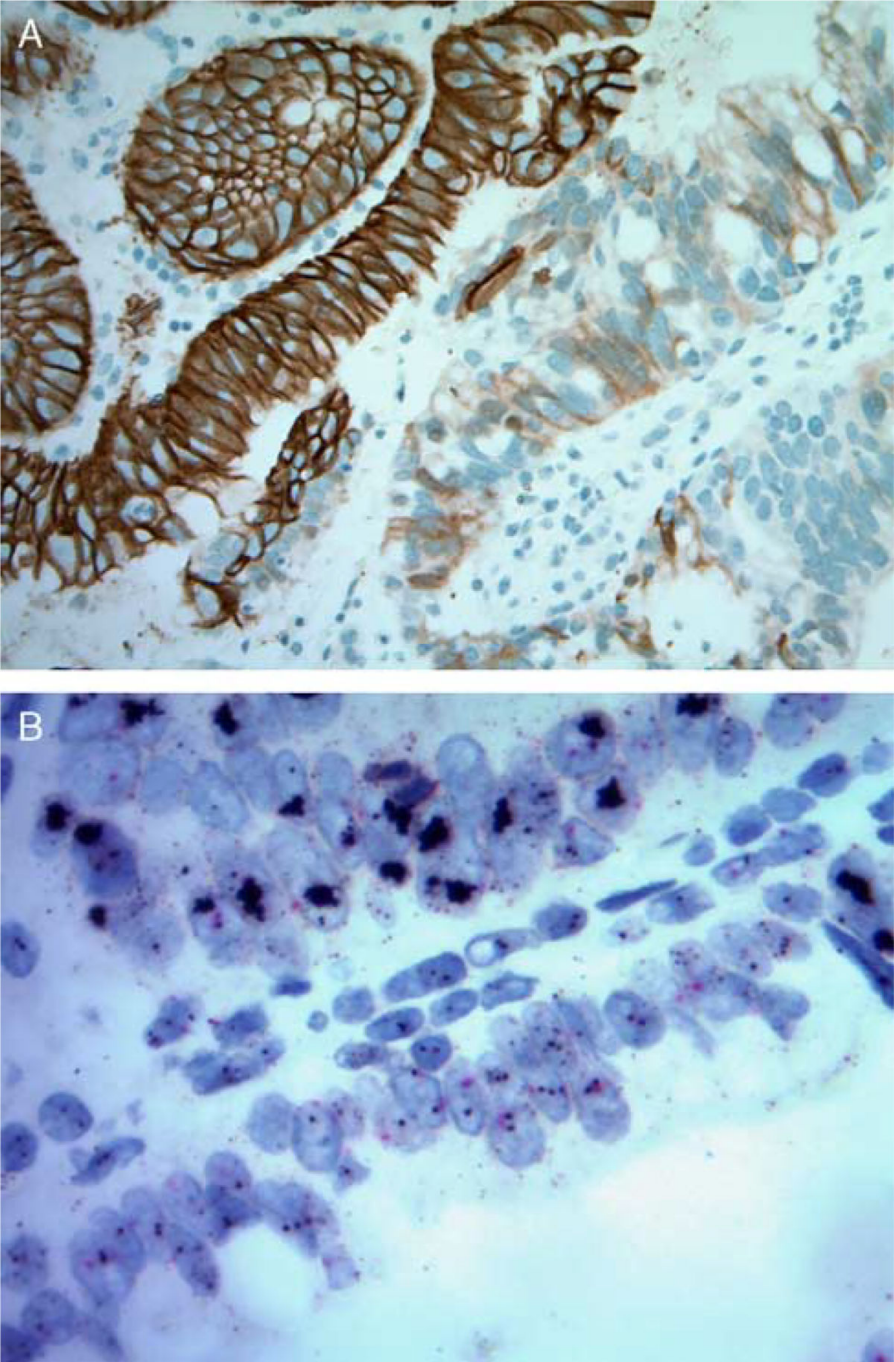
Whole-tissue analysis of Her-2 in a case of Barrett esophagus with high-grade dysplasia showing heterogeneity. Her-2 by immunohistochemistry, to the left positive results (3+), to the right is negative (1+) (A). Her-2 evaluation by dual in situ hybridization of the same area displaying amplification on the left and negative results on the right (B).

Significant tumor heterogeneity was observed in 6 cases. In 2 cases, tumor heterogeneity was observed in both IHC and DISH (cases 1 and 6) (Fig. 3). In 4 cases, tumor heterogeneity was observed only in IHC (cases 3, 4, 5, 9). In 2 cases, discrepant results between TMA and whole-tissue analysis were seen (cases 1 and 4). Both cases demonstrated tumor heterogeneity by IHC, DISH, or both. Results are shown in Table 3.

## DISCUSSION

In the present study, we examined Her-2 protein expression and *Her-2* gene amplification by IHC and DISH, respectively, in a subset of BE, LGD, HGD, and ICA samples. Similarly to previous reports, we found Her-2 overexpression in approximately 24% of ICA and, surprisingly, also in 28% of HGD. This was an unexpected finding that may have clinical implications. Interestingly, positive Her-2 DISH was also seen in 6% of LGD.

Despite surveillance programs for patients with BE, the incidence of esophageal adenocarcinoma has been increasing dramatically in the past 3 decades.^1^ Therefore, there is a strong need for markers of neoplastic progression to predict cancer risk in BE patients.

In a recent study, Fassan et al^14^ showed the early involvement of *Her-2* amplification and protein overexpression in both gastric and Barrett oncogenesis. In their study, Her-2 overexpression was found in only 4 of 25 LGD, 7 of 25 HGD, and 8 of 25 ICA. Similarly, we observed Her-2 protein overexpression and *Her-2* gene amplification in BE patients with HGD and ICA. Furthermore, *Her-2* gene amplification/protein overexpression and chromosome 17 aneusomy (polysomy) was not identified in NM and BE.

As in prior studies,^11,14–16^ we also observed Her-2 protein overexpression and *Her-2* gene amplification in a minority of LGD cases, suggesting the involvement of Her-2 in BE progression to dysplasia and carcinoma.

p53 alterations have been reported during the progression of BE.^17–19^ Aberrant p53 is accumulated in the nucleus of the neoplastic cells especially in HGD, and p53 IHC status has recently been shown to have diagnostic value and to predict neoplastic progression in patients with BE.^20^ In view of this finding, the recently reported association between Her-2 expression and p53 overexpression is not a surprise.^12^ Interestingly, Her-2 expression was associated with p53 overexpression especially in tumors that were at an early disease stage.^12^ Importantly, in our study, composed mostly of low-stage tumor cases (40 of the tumors were stage I and II), we found Her-2 overexpression in 28% and 24% of HGD and ICA, respectively, and gene amplification in 20% and 18% of HGD and ICA, respectively. These results are in contrast with those described in a prior study that focused on high-stage tumors.^13^ In their study, Yoon et al^13^ reported that only 17% of 713 esophageal adenocarcinomas were Her-2 positive and that Her-2 positivity was significantly associated with lower tumor grade, less invasive tumors, fewer malignant lymph nodes, and the presence of adjacent BE. However, others have reported a statistically significant correlation between Her-2 amplification/overexpression and poor prognosis in this patient population.^15^ In our study, a correlation with tumor prognosis could not be determined because the majority of tumors included in this cohort were of low stage. These findings suggest that evaluation of Her-2 expression may be useful in characterizing the evolution from BE to dysplasia and to ICA. The availability of a Her-2 blocking agent provides the possibility of alternative therapeutic approaches, as suggested by the results of preliminary studies. On October 2010, the FDA granted approval for trastuzumab in combination with cisplatin and a fluoropyrimidine (capecitabine or 5-fluorouracil) for the treatment of patients with Her-2-overexpressing metastatic gastric or gastroesophageal junction adenocarcinoma who did not received previous treatment for metastatic disease.^13^ Several ongoing trials are evaluating trastuzumab in esophageal and gastric cancer as first and salvage treatment in recurrent cancer. On the basis of our results, the prevalence of Her-2 expression and amplification in both HGD and ICA is relatively high, being present in approximately one fourth of the patients. The targeting of Her-2 in precancerous conditions should be considered and its utility should be tested in clinical trials. Further studies investigating the role of Her-2 on survival are also warranted.

Finally, tumor heterogeneity should be considered when testing small biopsies as well as in research studies using TMA technology. In these circumstances, determination of Her-2 status by IHC or DISH alone may result in a high false-negative and insufficient rate. Therefore, the combination of IHC and DISH testing as well as retesting on resection specimens in negative cases (if possible) is recommended.

## Figures and Tables

**TABLE 1.** Clinical Pathologic Findings of the Invasive Adenocarcinomas

Characteristics	
Age (range) (y)	41–83
Median	69
Sex	
Male	47
Female	3
Tumor size (cm)	0.2–7.3
Median (cm)	2.1
Tumor type (adenocarcinoma) (n)	
Well differentiated	5
Moderately differentiated	23
Poorly differentiated	22
Tumor stage (n)	
I	26
II	14
III	9
IV	1

**TABLE 2.** Comparison of Her-2/neu Levels by DISH (*P*<0.05)

	Tissue Type				
	NM	BE	LGD	HGD	ICA
Number	25	49	50	50	50
Her-2/Chr17 ratio <2	25	49	47	40	41
Her-2/Chr17 ratio ≥2	0	0	3	10	9

BE indicates Barrett esophagus; DISH, dual in situ hybridization; HGD, high- grade dysplasia; ICA, Invasive carcinoma; LGD, low-grade dysplasia; NM, normal.

**TABLE 3.** Results of the Her-2 Analysis by IHC and DISH Performed in a Subset of Cases to Evalúate Tumor Heterogeneity

	Case Number										
	1	2	3	4	5	6	7	8	9	10	11
TMA IHC	0	0	0	0	0	3+	0	3+	0	0	3+
TMA DISH	Not-Amp	Amp	Not-Amp	Amp	Not-Amp	IND	Amp	IND	Amp	IND	Amp
Tissue IHC	3+ (TH)	0	2+ (TH)	1+ (TH)	1+ (TH)	3+ (TH)	0	3+	1+ (TH)	0	3+
Tissue DISH	Amp (TH)	Amp	Not-Amp	Not-Amp	Not-Amp	Amp (TH)	Amp	Amp	Amp	Not-Amp	Amp
Diagnosis	HGD	HGD	ICA	ICA	LGD	HGD	ICA	ICA	HGD	BE	ICA

Scoring was as follows: negative = 0/1 +; equivocai = 2 +; positive = 3 +.

Amp indicates amplified; BE, Barrett esophagus; DISH, dual in situ hybridization; HGD, high-grade dysplasia; ICA, invasive carcinoma; IHC, immunohistochemistry; IND, indeterminate results; LGD, low-grade dysplasia; Not-Amp, not amplified; TH, tumor heterogeneity observed.
